# Buffalo Academy of Medicine

**Published:** 1900-07

**Authors:** Thomas F. Dwyer


### Society Proceedings.

### BUFFALO ACADEMY OF MEDICINE.

Reported by THOMAS F. DWYER, M. D., Secretary.

ANNUAL meeting held at the Academy rooms, Public Library Building, Tuesday, June 12, 1900. The meeting was called to order at 9 p.m. by the president, Dr. Matthew D. Mann. The minutes of the last annual meeting were read and approved. The secretary of the Academy and council presented the following report, which, on motion, was received and ordered spread on the minutes.

### ANNUAL REPORT OF THE SECRETARY.

*To the Academy of Medicine:*

During the past fiscal year eleven applications for membership have been received and favorably acted upon, of which nine were resident Fellows and two non-resident Fellows. Thirteen members have been dropped for nonpayment of dues and three resignations have been received and accepted during the year. Two members who had been dropped for nonpayment of dues were readmitted to membership. I am pleased to be able to again report that the membership of the Academy has not been decreased by death since the last annual meeting. The program for the session 1899-1900, together with a list of Fellows in good standing was printed and a copy sent to each member.

On December 5, 1899, a new section was added to the Academy, the Section of Ophthalmology, Otology, Rhinology and Laryngology.

The Academy parlors in the Palace Arcade, so long occupied by the Academy, were vacated May 1, 1900, and the rooms of the Buffalo Society of Natural Sciences, Public Library Building, have been leased for the present year. Four regular meetings were held and entertained in turn by the sections of the Academy: Section of Obstetrics and Gynecology, June 27, 1899, at which Dr. Wm. P. Spratling, Craig Colony, Sonyea, N. Y., was to have read a paper, but telegraphed at a late hour that it would be impossible for him to be present. Section of Medicine, September 12, 1899, The medical aspects of crime, Dr. Daniel R. Brower, Chicago, Ill. Section of Surgery, December 5, 1899, The value of normal salt solution in surgery, Dr. Chauncey P. Smith; Hypertrophy of the prostate, Dr. Charles F. Durand; Desiccated suprarenal capsule in acute coryza, Dr. Frederick H. Millener. Section of Pathology, March 20, 1900, The present status of recent investigations on cancer, Dr. H. R. Gaylord.

The meetings of the Academy have been attended as follows: at the June meeting 20 were present; September meeting, 61; December meeting, 21; and the March meeting, 67, an average attendance of 42¼.

The council of the Academy held eight meetings with an average attendance of 10⅝ members. Respectfully submitted,

THOMAS F. DWYER, Secretary.

The report of the treasurer was read by Dr. EUGENE A. SMITH. On motion report was received and referred to an auditing committee, consisting of the trustees. The report of the trustees was read by Dr. Marcel Hartwig. It was moved and seconded that the report be received and referred to a committee of two to audit. The chair appointed as such committee Drs. Eli H. Long and J. C. Clemesha. This committee later reported having audited the accounts and moved that the report of the trustees, with the recommendations, be adopted. Carried.

Reports were received from the secretaries of the Sections of Medicine, Surgery, Obstetrics, Pathology and Ophthalmology, and on motion were received and ordered filed.

The following proposed amendments to the by-laws were presented by Dr. S. Y. HOWELL:

BY-LAW I.

Sec. 1. The stated meetings of the Academy shall be held during the last week of September, November, January, March and May, on such days as the council may determine.

Sec. 2. The annual meeting for the election of officers, the reading of annual reports, and the transaction of other business, shall be held on the first Tuesday of June.

Sec. 3. The meetings of the sections shall begin the first week in October, and end with the fourth Tuesday in May. Each section shall meet at least once each month during this time, except when called upon to entertain the Academy at one of its stated meetings.

Section 3 shall become section 4.

Section 4 shall become section 5.

It was moved and seconded that a committee on revision of the constitution be appointed. It was moved as an amendment that the matter of revision of the constitution be referred to the council. Carried.

The retiring president, Dr. Matthew D. Mann, then read his annual address, the subject of which was Puerperal Infection.

Upon invitation the address was discussed by Drs. P. W. Van-Peyma, M. A. Crockett, L. Schroeter, H. D. Ingraham, I. P. Lyon, J. V. Woodruff, J. H. Pryor and S. Y. Howell.

Dr. Marshall Clinton, on behalf of the tellers, reported the result of the election as follows: President, Dr. Marcel Hartwig; treasurer, Dr. Charles S. Jewett; trustee, Dr. Joseph W. Grosvenor.

The newly elected president, Dr. Marcel Hartwig, was escorted to the chair and presented to the Academy by Dr. Mann. Dr. Hartwig thanked the members of the Academy for the honor they had conferred on him in selecting him as the presiding officer for the ensuing year.

He offered a prize of $50 to be given for the best paper presented at the meetings during the coming session, the council to make the necessary arrangements.

Attendance, 51. Adjourned at 11 p. m.

---

### *Section on Surgery, April 6, 1900.*

Reported by MARSHALL CLINTON, M. D., Secretary.

The meeting being called to order by the Chairman, Dr. J. Henry Dowd, the minutes of the previous meeting were approved.

Dr. Paul Thorndike, of Boston, by invitation, read a paper entitled,

### NOTES ON PROSTATECTOMY.

He said it was not until the publication of Mr. Arthur McGill's cases in 1888, and his own and his colleagues (of the Leed's Infirmary) communications to the British Medical Association in 1889 that the surgical world awoke to the possibility of attempting a radical operation for the removal of obstructing prostatic masses. He proposed at this time simply to invite attention to certain points connected with radical prostatic operating.

The present status of the treatment of prostatics may briefly be summarised as: (1) the palliation of the condition by catheter as long as the patient so can be kept comfortable; (2) then attempt to alter the existing obstruction by various operative procedures. These latter are: (*a*) the removal of one or both testicles; (*b*) the ligature of one or both vasa deferentia; (*c*) the removal of parts of the prostatic growth by incision, enucleation, or electric heat; or, (*d*) by a complete enucleation of the growth by suprapubic or perineal routes, or both combined.

He passed the tentative measures with brief comment and proceeded to discuss the radical operation, stating that the suprapubic operation with perineal drainage remains the one of choice with most surgeons. In McGill's operation the bladder is opened above the pubes, the mucous membrane covering the projecting portions of the prostate is cut through, and obstructing portions are removed by enucleation by the finger, forceps, or both. Fuller, of New York, has modified this technique by making a small opening in the mucous membrane covering the growths and enucleating them through this with the finger; he also opens the membranous portion of the urethra and drains through the perineum.

Thorndike then described the operation of Alexander and Nicoll and reviewed the statistics of all the several methods. Continuing he said, if a partial operation is done there is chance of recurrence of the obstruction; whereas, if the complete operation is done there is no recurrence. Prostatectomy is a serious operation with a mortality result of 15 or 20 per cent. He favors the Alexander operation. Alexander opens the bladder over the pubes, and then places the patient in the lithotomy position with a grooved staff passed into the bladder through the urethra. He then opens the membranous urethra, by a median incision from just behind the bulb back to the apex of the prostate. The staff is then withdrawn, two fingers of the left hand are passed into the bladder through the suprapubic wound, which serve to press the prostate downward into the perineal wound. The enucleation is then done with the forefinger of the right hand through the perineal opening.

Dr. Thorndike saw Alexander perform his first operation by this method a few years ago. Lately, he had seen him operate on two more cases and was in each instance impressed with the ease and rapidity of the procedure. He, himself, had operated twice in this manner, and these had further confirmed his good opinion of the method. Objection to it has been made because it cannot be of use in cases where the third lobe projects much into the bladder. One of his cases was of this kind and it was interesting to realise, he said, how easily the third lobe could be pushed down and enucleated, after the more easily accessible lateral lobes had been removed. It was available, too, in small hard prostates, for such an one he had seen Alexander remove with the greatest ease.

His object in presenting the subject was to create interest in this method of operating which he was convinced had a place, and that it completely meets the requirements of the cases in which it can be appropriately done.

Concluding he said: "We have two present duties; first, to teach the general medical public the limitations of catheter treatment and the necessity of letting the surgeon be his own judge as to the proper time for interference, as well as to judge of the technical character of that interference, to teach them that prostatics should be made comfortable and kept so by radical operation, early in the disease, if other means fail or cannot be properly instituted.

"Second, to teach ourselves the possibilities which these new operations are opening up to us and to give ourselves an experience which will justify us, as it most certainly is going to do, in offering a radical operation to persons so early in the progress of the diseases, that renal complications will be as rare a cause of death as shock, hemorrhage, and sepsis are already coming to be."

DISCUSSION.

Dr. W. H. Heath said patients will not be operated unless in extremis and then we have an organism in the poorest possible condition for any work on the genito-urinary tract. He agreed with Dr. Thorndike in his views of this trouble and thinks that large succulent prostates particularly should be removed by the suprapubic route. He reported a case on which a Bottini's operation had been performed and the patient was able to urinate at once. He wished Dr. Thorndike's opinion on this procedure.

Dr. H. Mynter requested some information in regard to the pathology of the prostate and reported five recoveries following simple perineal section.

Dr. E. J. Meyer reported that while in Berlin last summer Sidelow reported Bottini's operation as very unsatisfactory in the hands of the German surgeons.

Dr. B. H. DAGGETT said no operation on the prostate or bladder for the relief of enlarged prostate is successful if residual urine remains afterward. Prostatectomy should be done early.

Dr. THORNDIKE, in closing the discussion, said the reason that palliative measures were so much in vogue is that they are so easy and make the patient comfortable for the time being. He thinks that as the profession generally becomes more familiar with this radical procedure and appreciates the ease with which it can be performed, with practically no death-rate, and perfect recovery with restoration of bladder function, that palliative measures will be abandoned for the choice of this operation. In regard to Bottini's operation he considers it both unsurgical, unscientific and unsafe.

On invitation of Dr. Parmenter, Dr. Thorndike removed an enlarged prostate at the Buffalo General Hospital clinic and the time spent in entering the bladder and enucleating the prostate was eleven minutes.

Attendance, 47.

---

*Section on Ophthalmology, Otology, Rhinology and Laryngology, April 16, 1900.*

REPORTED BY RICHARD H. SATTERLEE, M. D., Secretary.

The meeting was called to order at 8.50. Dr. W. SCOTT RENNER took the chair.

Minutes of last meeting were read and approved.

Dr. B. H. GROVE presented two papers: Notes on the Advancement of the Ocular Muscles (see page 879); Some Thoughts Suggested by the Local or Medicinal Treatment of Cataract (see page 881.)

DISCUSSION.

Dr. A. A. HUBBELL—I quite agree with Dr. Grove in his remarks on advancement. The operation should not only be performed for secondary deviations following tenotomy, but for the correction also of primary squint. I think, however, that convergent squint should be treated by correcting refractive errors, and when this is done early, there is often no need of operation. This method has been so satisfactory that I have seldom had occasion in many years to operate. I sometimes operate for exophoria or hyperphoria, but I do so only when other treatment has failed. When I do operate for heterophoria, I find it necessary to do more than to simply buttonhole the tendon, unless by this is meant the division of the tendon up to its fascial extension. I am not able to get any effect until the essential part of the tendon is severed.

When a slight advancement is indicated, I think Stevens's method is by far the best. In advancement for squint, I have never practised anchoring the sutures in the sclera, as is done by Dr. Grove, the subconjunctival and episcleral tissues always proving sufficient. In passing the needle from within outward through the muscle as far back as is necessary, I also include Tenon's capsule and the conjunctiva, the muscle having been thoroughly loosened from the ball for quite a distance back and also to each side, and held by an ordinary fixation forceps. In tightening the sutures, I prefer to have a slight convergence. I remove the sutures in three days, believing that the adhesion of the parts to their new surroundings is as firm as at a later period. The stitches, even at this time, are more or less loosened, and serve no further purpose whatever, and are only sources of irritation.

As regards the dissipation of cataracts by medicines, rubbing, electricity, and the like, I do not believe it is ever done. For centuries, medicines innumerable—vapors, ethers, drugs, both applied to the eye and taken internally—have been used. From time to time success has been claimed, but such success has not rewarded others. The healthy lens is made up of cells and laminæ, most delicately arranged, and these become broken up or disintegrated, and I do not believe any medicine or agency can restore them to a normal condition after they have once lost their integrity and transparency. I congratulate Dr. Grove in his exposure of the latest "cure" of cataract by medicine. I expect, however, that, while Dr. Grove will reach some who will heed his advice, there will be very many others who will continue to patronise these "adventurers," for operations are always dreaded, and people will try any measure, no matter what the profession think of it, which promises relief without an operation.

Dr. Lucien Howe—Dr. Grove seems to consider the question of age as the standard for determining the time for operation. I think, however, that the degree of vision is more important. If this be good in each eye the longer we wait the better; if the vision be poor or decreasing, then we should operate.

As for anchoring the stitch, that is an important matter and unless care is exercised, we are apt to turn the eye too far up or too far down, especially when the muscle and fascia is drawn up in a lump. After trying many methods, I have found that three sutures give better results as to position and leave less scar.

As for remedies for the cure of cataract, I try them all whether advertised or not. The last mentioned remedy is absolutely useless. I learned a good deal of a noted quack institution in the north eastern part of this state. A patient of mine—a prominent woman—went there twice. She came back extolling some drops that were given her. I put some of them in a normal eye and they dilated the pupil. It is not impossible that we may find a method of curing cataract without operation. Whether it will be by cataphoresis or what I do not know.

Dr. BURROUGHS—I wish to mention in this connection Dr. Kalish's method—equal parts boracic solution and glycerine. The lids after instillation to be rubbed in just such a way for ten to fifteen minutes. A relative of mine having lenticular opacities was treated by a general practitioner by the Kalish method and the development of the cataracts were certainly stopped. Later I sent a traumatic cataract to Dr. Kalish, but he refused to treat the case.

Dr. E. E. BLAAUW—I would like to ask the last speaker if he would try the Kalish method?

Dr. BURROUGHS—I should try it.

Dr. BLAAUW—I should like to know if you have ever noticed any change with the ophthalmoscope after treatment by this method in the case of your relative.

Dr. BURROUGHS—Most certainly, the periphery of the lens was decidedly cleared up.

Dr. BLAAUW—If I had a child with squint, I should not wait until it was thirteen or fourteen years of age. We should operate on both eyes. I do not think tenotomy of the interni should be done. I would prefer advancement of the externi. I have read about Dr. Ranney's button-holes. I don't think you get any result from buttonholes. It is impossible to graduate the effect you get later on. Absorption of cataract is an exceedingly interesting question. We have all seen where cataract went. So far there was stationary, then tended to clear up of itself. For example, some traumatic cataracts. Diet will accomplish much in cataracts due to diabetes or albuminuria.

Dr. ARTHUR G. BENNETT reported a case, 75 years of age, of amblyopia, associated with divergent strasbismus and a high degree of hyperopia. Covering the good eye with ground glass, the sight was brought to $\frac{5}{22.5}$ with Snellen vj.

Also a case where solution of atropia had been dropped in the eye for twenty-five years for poor vision, owing to lenticular opacities. Complete atrophy of iris. Extraction of cataract was followed in five days by severe iritis. Needling of membrane was followed by severe iritis. The final result was vision $\frac{20}{70}$.

---

### *Section on Pathology, April 18, 1900.*

REPORTED BY FREDERICK H. MILLENER, M. D., Secretary.

The eighth regular meeting of the Pathological Section for the season was held on Tuesday, April 18, 1900.

The meeting was called to order by the Chairman, Dr. Bennett, at 8.50 p.m., twenty-five Fellows being present.

The minutes of the preceding meeting were read and approved.

The paper of the evening was the Gynecological Mistakes and their Pathological Importance, by Dr. HERMAN E. HAYD (see p. 871).

The discussion was opened by Drs. Crockett and Mann.

Dr. M. A. Crockett stated the paper covered a wide field, and that he thought that they should divide the gynecological mistakes or errors into three classes; first, those mistakes where the disease is an extension of some other disease, notably that of the rectum; second, those cases due to lack of skill in diagnosis and lack of knowledge on the part of the physician. As an instance, he cited a case where ergot had been used to expell a uterine fibroid; the third is a very important class, in which there is a local lesion present and the general symptoms. The patient has anemia, backache, etc., and, save for the slight lesion, the case can only be cured by studying and treating all parts involved.

The mistakes by the general practitioner in gynecology are no greater than are made in different parts of the body by other specialists. A great many mistakes come from general practitioners, who emphasise the local lesions.

Dr. Matthew D. Mann stated that Dr. Hayd's paper was a very timely one as he had gone over the subject very carefully. Dr. Mann spoke of bladder and rectal troubles, which were sometimes overlooked, and cited a case of a woman, who was pregnant and had bearing down pains. A physician was called and decided to bring on an abortion. The real trouble being a gonorrheal infection of the bladder.

He agreed entirely with Dr. Hayd with regard to simple endometritis being generally due to constitutional conditions, and that it was wrong to make local applications to the uterus; we should treat the patient generally. He considered the matter of curetting the uterus very much overdone, and that a great deal of harm is being accomplished by irritating it without any good. He also pointed out a mistake he had seen made several times, namely, the anterior surface of the spinal column and aorta being mistaken for a tumor. Another common error physicians make in making a rectal examination is feeling the uterus and mistaking it for a tumor.

Dr. Grosvenor related an experience of a young and ambitious physician, who found a young woman very willing to be operated upon; he examined the case very carefully and found a tumor, as supposed, and invited some physicians to be present at an operation; in looking for the tumor under chloroform, it had mysteriously disappeared. He referred to an error of an experienced physician, who mistook a fibroid ovarian tumor, called physicians to witness the operation, opened the abdominal cavity and instead found hydatids of the liver.

Dr. Hayd, in closing the discussion, said that he was pleased that the paper met with approval, as he had endeavored to make it profitable. Dr. Hayd also stated that mistakes are not always those of ignorance, but also those of dishonesty.

Meeting adjourned at 10.20 p.m.

*Section on Medicine, May 8, 1900.*

REPORTED BY J. C. CLEMESHA, M. D., Secretary.

The eighth regular meeting of the Medical Section, Buffalo Academy of Medicine was held May 8, 1900, at the Buffalo Public Library Building. The meeting was called to order at 8.45 p.m. In the absence of Dr. Long, Dr. M. D. MANN acted as chairman.

After reading the minutes of the previous meeting Dr. H. R. HOPKINS was nominated and elected chairman of the section for the ensuing year. Dr. JULIUS ULLMAN was nominated and elected secretary.

Dr. ULLMAN reported the care of a child with influenza having four distinct pneumonic attacks, otitis media purulenta, colitis, marasmus; recovery.

Dr. BENEDICT read a paper on Typhilitis and scolecises, which was discussed by Dr. Grosvenor and Dr. Dunham.

Madame Mankel gave an instructive address upon the dress of children, noting defects and pointing out improvements in construction.

Meeting adjourned at 10.50 p.m. Members present, 27.

